# *Phoenix* Is Required for Mechanosensory Hair Cell Regeneration in the Zebrafish Lateral Line

**DOI:** 10.1371/journal.pgen.1000455

**Published:** 2009-04-17

**Authors:** Martine Behra, John Bradsher, Rachid Sougrat, Viviana Gallardo, Miguel L. Allende, Shawn M. Burgess

**Affiliations:** 1 National Human Genome Research Institute, National Institutes of Health, Bethesda, Maryland, United States of America; 2 National Cancer Institute, Bethesda, Maryland, United States of America; 3 National Institute of Child Health and Human Development, Bethesda, Maryland, United States of America; 4 Center for Genomics of the Cell, Facultad de Ciencias, Universidad de Chile, Santiago, Chile; University of Pennsylvania School of Medicine, United States of America

## Abstract

In humans, the absence or irreversible loss of hair cells, the sensory mechanoreceptors in the cochlea, accounts for a large majority of acquired and congenital hearing disorders. In the auditory and vestibular neuroepithelia of the inner ear, hair cells are accompanied by another cell type called supporting cells. This second cell population has been described as having stem cell-like properties, allowing efficient hair cell replacement during embryonic and larval/fetal development of all vertebrates. However, mammals lose their regenerative capacity in most inner ear neuroepithelia in postnatal life. Remarkably, reptiles, birds, amphibians, and fish are different in that they can regenerate hair cells throughout their lifespan. The lateral line in amphibians and in fish is an additional sensory organ, which is used to detect water movements and is comprised of neuroepithelial patches, called neuromasts. These are similar in ultra-structure to the inner ear's neuroepithelia and they share the expression of various molecular markers. We examined the regeneration process in hair cells of the lateral line of zebrafish larvae carrying a retroviral integration in a previously uncharacterized gene, *phoenix* (*pho*). *Phoenix* mutant larvae develop normally and display a morphologically intact lateral line. However, after ablation of hair cells with copper or neomycin, their regeneration in *pho* mutants is severely impaired. We show that proliferation in the supporting cells is strongly decreased after damage to hair cells and correlates with the reduction of newly formed hair cells in the regenerating *phoenix* mutant neuromasts. The retroviral integration linked to the phenotype is in a novel gene with no known homologs showing high expression in neuromast supporting cells. Whereas its role during early development of the lateral line remains to be addressed, in later larval stages *phoenix* defines a new class of proteins implicated in hair cell regeneration.

## Author Summary

By screening for regeneration deficient zebrafish mutations, we identified a zebrafish mutant line deficient in a highly specific regeneration process, the renewal of hair cells in the lateral line. Although this organ is specific to fish and amphibians, it contains essentially the same mechanosensory cells (the hair cells) that function in the ear for sound and balance detection in all vertebrates. Mammals are unusual vertebrates in that they have lost the ability to regenerate functional hair cells after damage by sound or chemical exposure. All other vertebrates retain their ability to regenerate their hair cells after damage, but this process is not well understood at the molecular level. The retroviral insertion linked to the *phoenix* mutation is in a new gene family class that is specifically required for the supporting cells to enter into mitosis after hair cell damage. What is particularly unusual about this mutation is that it appears not to affect the normal development and differentiation pathways, but only seems to affect the cells' post-differentiation regeneration.

## Introduction

During development of the vertebrate inner ear, a subset of neuroepithelial cells specialize to give rise to hair cells and supporting cells [1]–[3]. These two cell populations assume distinct and complementary functions. The hair cell is a highly differentiated mechanoreceptor cell, transducing sound waves in the cochlea or acceleration and head movements in the vestibular organ, into electrical signals [4],[5]. The supporting cells provide cohesive support [6],[7] and have secretory functions [8],[9]. More importantly, they have been clearly implicated in the addition of new hair cells, both during normal growth and during restoration of the sensory epithelium after damage in various animal models [10]–[14]. Thus, among supporting cells, there exists a tissue-specific population of progenitor cells. However, in mammals, this “stem cell like” property is lost shortly after birth in most neuroepithelia of the inner ear [15]. With the exception of some limited regeneration in a sub-region of the vestibular organ [16],[17], post natal hair cell loss is permanent and irreversible. Consequently, a large majority of deafness cases in humans are linked to absent or damaged hair cells. To restore the regenerative capacity of supporting cells is an obvious therapeutic aim, but our understanding of the regenerative process is incomplete. Because birds, amphibians, reptiles, and fish have retained the ability to regenerate hair cells [14], [18]–[25] they provide opportunities to find genes involved in the regeneration process and its maintenance.

Fish and amphibians have an additional organ related to the inner ear called the lateral line, which is used to detect water currents [26]–[28]. It is a superficial organ running along each side of the body which consists of stereotypically distributed patches of neuroepithelium, called neuromasts. These are discrete organs made of hair cells that project into the aqueous environment and supporting cells that surround them. In the zebrafish embryo, the lateral line neuromasts first appear in the head approximately 2 days post fertilization (dpf) and in parallel begin to develop along the entire length of the trunk and tail [29]. Hair cells are fully functional by 4dpf [30] and as the larva grows into an adult, additional neuromasts are continually added to the embryonic pattern [31],[32]. In adult fish, newly formed neuromasts are thought to originate from preexisting ones, with supporting cells budding off and migrating to their new locations [33]. Additionally, this sensory organ is known to continuously replace hair cells in larvae and adults [34]. The neuromasts are also able to replace hair cells after all existing ones have been have been destroyed [35],[36]. Several waterborne agents have been used to eliminate hair cells from neuromasts, including aminoglycosides, platinum-based drugs, and metal ions. Copper is a potent ototoxic agent, killing hair cells in the lateral line, as early as 3dpf [37]. After 5dpf, the hair cells also become sensitive to antibiotics of the aminoglycoside family, in particular to neomycin, presumably coinciding with their functional maturity [38],[39]. At all time points, regeneration of hair cells has been documented, mainly resulting from the division of supporting cells and subsequent differentiation into new hair cells [14], [40]–[44].

We utilized an *in vivo* assay using both copper and neomycin to follow regeneration of hair cells in neuromasts of 6dpf to 8dpf larvae. Using this assay, we characterized two allelic mutant lines generated by retroviral insertion [45], which we have named *phoenix* (*pho*). Homozygous mutants did not display obvious morphological or behavioral phenotypes and developed a functional lateral line as ascertained by FM1-43 incorporation. However, when *phoenix* larvae were treated with either copper or neomycin, they showed a strongly reduced regeneration of the hair cells. In parallel, we demonstrated that another form of regeneration, the growth of the tail after amputation, was not affected in the *phoenix* mutants, arguing that the regeneration defect was specific to hair cells.

We show that, the number of supporting cells was not significantly different before or after copper and neomycin treatments in neuromasts of wild-type versus *phoenix* mutant larvae. Furthermore, we monitored cell death over the time course of our assay in treated wild-type and mutant neuromasts and did not find significant differences in supporting cell death. Strikingly, we found that the proliferation rate in this progenitor cell population was strongly reduced in mutants. Thus, impaired proliferation is tightly correlated to the observed deficit of regenerated hair cells in the *phoenix* mutant.

We characterized the gene carrying the retroviral integration linked to the phenotype, and find that it is a novel gene, with no previously described homologs. Expression at 3-4dpf is upregulated in the supporting cells of the neuromasts. Thus, *Phoenix* is the first documented member of a novel gene family, which has an important role in the regeneration of hair cells in the lateral line.

## Results

### Monitoring hair cell regeneration in the lateral line of zebrafish mutant lines

**Figure 1:**
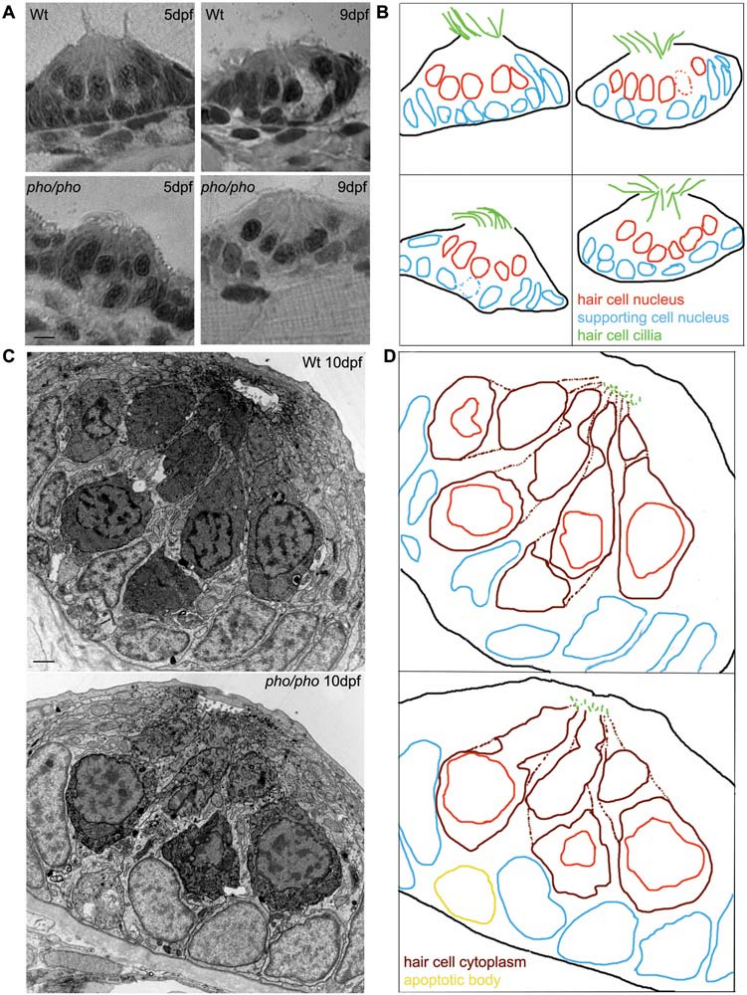
The development of the neuromasts in the lateral line is normal in the *phoenix* mutant larvae. (A) Semi-thin sections showing wild-type (top panels) and mutant neuromasts (bottom panels) in 5dpf (left panels) and 9dpf (right panels) larvae. (B) *Camera lucida* drawing for each section, highlighting the hair cells nuclei (red) and their cilia (green) and the supporting cells nuclei (blue). (C) Ultra-thin sections viewed by Electron Microscopy (EM) of a wild-type (top panel) and a mutant (lower panel) neuromast in 10dpf larvae. The hair cells stain darker than the supporting cells. (D) *Camera lucida* drawings of the EM sections, highlighting the hair cell nuclei (red), cell bodies (dark red), and cilia (green). The nuclei of the supporting cells are highlighted (blue). One apoptotic body was visible in (yellow). – 5 microns in (A) and 1 micron in (C).

We have utilized an assay to monitor hair cell regeneration in the lateral line of 5dpf zebrafish larvae, using transient exposure to copper sulphate (10mM) or neomycin (200mM) dissolved in the water. Our intention was to test for defective hair cell regeneration from a collection of mutant lines previously identified in a retroviral integration screen [45]. The major advantage of using retroviral constructs, over chemicals like ENU, as a mutagenic agent is the rapid identification of the mutated gene. This allows the spatio-temporal expression of the mutated genes to be compared to the observed phenotypes, facilitating selection of the mutants of interest. We reasoned that mutant larvae which develop a normal and functional lateral line and that exhibit expression of the mutated gene in neuromasts would be good candidates to test for defects in the regeneration of the hair cells. We found such a mutant line, which we called *phoenix*. Homozygotes displayed no behavioral defects (response to sound or mechanical stimulation was normal, data not shown) or visible morphological abnormalities until 5dpf, when the swim bladder failed to inflate. Later, around 7 to 8 dpf, mutants display necrosis in the liver, and death ensues at approximately 14 dpf. Because the gene was expressed in the lateral line neuromasts (see below), we performed a detailed assessment of this organ in 2 to 12 dpf larvae (Figure 1). Semi-thin sections through neuromasts (Figure 1A) of *phoenix* mutants (lower panels, 5dpf left panel and 9dpf right panel) were indistinguishable from wild-type larvae (upper panels). *Camera lucida* drawings (Figure 1B) of each section show the cilia (green) of hair cells and their nuclei (red) and the nuclei of the supporting cells (blue). Likewise, ultra-thin sections observed by electron microscopy (EM) (10dpf larvae shown in Figure 1C) did not present obvious differences between wild-type (top panel) and mutant neuromasts (lower panel). *Camera lucida* drawings (Figure 1D) show, like in Figure 1C, the cilia (green) of hair cells and their nuclei (red) and the nuclei of the supporting cells (blue). The cytoplasm of hair cells is outlined in dark red and an apoptotic body is shown in yellow in the lower panel. Note that apoptotic bodies, as the one present in the mutant, were found at the same rate in untreated wild-type and mutant sections, being probably products of the regular turnover of the supporting cells, which has been described previously [34]. We further observed wild-type and mutant neuromasts in three different transgenic backgrounds (Figure S1). As shown in live images, these lines express GFP in all the cells of the neuromast in the *cldnB::GFP* line [46] (Figure S1A), in the hair cells in the *pou4f3::GFP* line [47] (Figure S1B) or in a subset of supporting cells in the *ET20::GFP* line [48] (Figure S1C). *Camera lucida* drawings were added to each panel, for better illustration of the live images. To test the functionality of the hair cells in wild-type and *phoenix* mutant larvae, we used the well-described technique of monitoring the absorption of the vital dye FM1-43 [49]. We imaged live wild-type and mutant larvae in the *cldnB::GFP* line background (Figure S1A, GFP in green in first and third columns, FM1-43 in red in second and third columns. *We added camera lucida* drawings of the merged images in the fourth column). Again, we did not find a significant difference between the absorption of the dye in wild-type (top) and mutant (bottom) hair cells, nor did we see any significant observable structural differences. Therefore, although the gene is expressed in the lateral line, its initial development appears unaffected in mutant larvae.

### Mutations in the *phoenix* gene are linked to the regeneration phenotype

**Figure 2:**
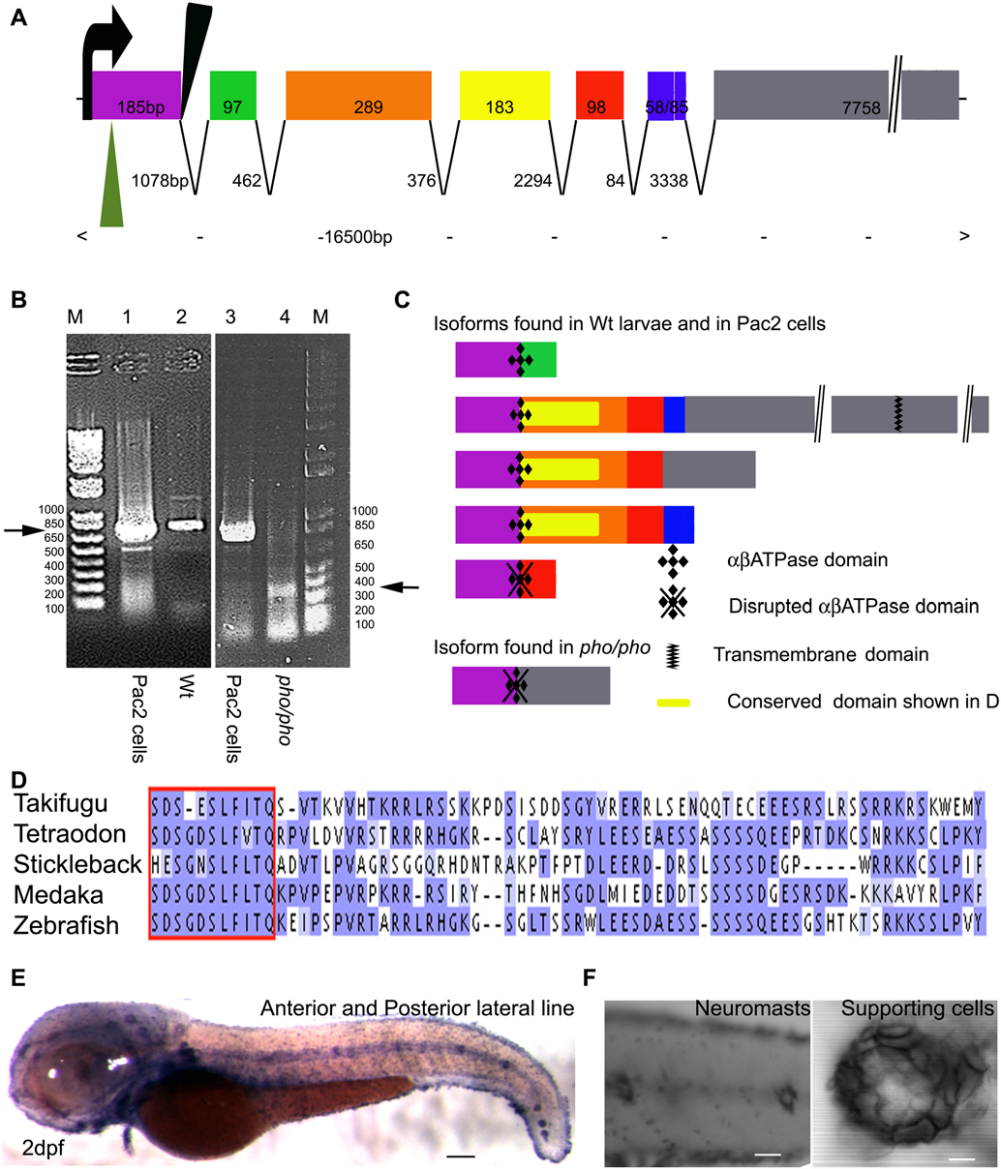
*Phoenix* is a gene without obvious homologs expressed in the supporting cells of the lateral line. (A) The coding region of the *phoenix* gene spans approximately 16.5kb with seven spliced exons. Exon 7 is an unusually long ORF of 7758bp. The retroviral integration in the *zm* allele (green triangle) is in the beginning of the first exon, probably leading to a null allele. In the *hi43* allele, the retroviral integration (black triangle) is adjacent to the first splice donor site inducing an aberrant spliced mRNA (see [B] and [C]). The size of exons and introns are indicated. (B) RT-PCR on total RNA extracts of the zebrafish cell line PAC2 (lanes 1 and 3) and 7dpf wild-type (lane 2) and *hi43* mutant allele (lane 4) larvae. The expected band of 835bp (left ->) was detected in the PAC2 cell line (lane 1 and 3) and in the wild-type larvae (lane 2). However, in the *hi43* mutant lane there is a new 368 bp band representing an aberrant splice event from exon 1 to exon 7 (right ->). (C) We identified five splicing variants from wild-type larvae and the PAC2 cell line and one in the *hi43* mutant allele larvae. In the mutant mRNA, the ab ATPase domain (plusses) is disrupted by the aberrant splice (diamonds). A putative transmembrane domain is found in exon 7 (zig-zag bar). The location of the conserved domain described in (D) is shown (yellow rectangle). (D) Alignment that shows the region of homology for putative *phoenix* genes in four other fish species. One particular stretch seems to be strongly conserved in all fish species, the short SDS- X(3)-SLF-[ILV]-TQ sequence (red box). The remaining sequence for all predicted proteins shows little to no significant identity, beyond enrichment for proline and lysine residues. (E) *In situ* hybridization with an antisense probe directed against the *phoenix* gene. At 2dpf the expression was found in all the neuromasts of the anterior and posterior lateral line. (F) Higher magnification of two trunk neuromasts (left panel) and a single neuromast (right panel), showing the typical ring-like staining restricted to the supporting cells. – 100 microns in E, 50 microns in (F, left panel) and 10 microns in (F, right panel).

The first allele (*hi43*) of the *phoenix* mutant line was generated by retroviral mutagenesis as previously reported [45]. The genomic location of the retroviral integration was isolated and used to genotype the offspring to maintain the mutant line through numerous generations [45]. We identified a BAC spanning the genomic locus from a zebrafish BAC library and sequenced it in its entirety. Using GENSCAN [50], we predicted the genomic structure of *phoenix* (Figure 2A). The gene spanned ∼16.5 kb with seven exons, the final predicted exon being unusually long as it was comprised of 7758bp with the entire exon consisting of an open reading frame. The size of the exons and introns is indicated in Figure 2A. The retroviral integration in *hi43* (black triangle) landed in the first exon and was adjacent to the first splice donor site.

To confirm that the retroviral insertion disrupted the surrounding gene, we designed primers to perform RT-PCR on total RNA extracts of 7 dpf wild-type and mutant larvae and on the zebrafish tissue culture cell line Pac2, which also expressed the gene [51]. The expected 835 bp RT-PCR product was seen (Figure 2B, left arrow), in wild-type larvae (lane 2), and in the Pac2 cell line (lane 1 and 3), but was absent in the mutant larvae (lane 4). However a shorter 366 bp product was generated in the mutant larvae exclusively (Figure 2B, right arrow). We cloned and sequenced all of the observed PCR fragments. In *pho* mutants, aberrant splicing occurred, fusing exon 1 to exon 7 and causing a deletion and a frame-shift truncation of the presumptive protein product (Figure 2C). Additionally, the aberrant splicing event resulted in an arginine replacing the last serine in a predicted abATPase signature, therefore potentially abrogating the putative enzymatic activity (Figure 2C).

To further prove that the deficient regeneration of the hair cells in the lateral line was caused by a mutation in the *phoenix* gene, we acquired a second commercially available allele (Znomics, Inc. line: ZM_00003486). This mutant line carried a retroviral insertion in the first exon (75bp downstream of the ATG) of the *phoenix* gene (green triangle Figure 2A). The insertion of the provirus in this position most probably led to a null mutation. All of the phenotypes observed in the original *hi43* allele were present in the second recovered mutation and all subsequent experiments were performed in both allelic mutant lines.

We did not attempt to phenocopy the regeneration phenotype using morpholino injection, as our observations started at 6dpf. This time-point is beyond the time-window of efficacy of morpholinos, which get diluted over time after the numerous cell divisions occurring in the growing embryo/larva.

Taken together, our data strongly indicate that we have correctly identified the genetic defect responsible for the observed phenotype.

### *Phoenix* is a novel gene encoding a large protein with low sequence complexity and which is strongly expressed in neuromast supporting cells

Using RT-PCR, we identified several alternatively spliced variants of the *pho* gene (Figure 2C). We compared the different isoforms against various RefSeq databases using BLAST [52]. The best predicted homologs in other species for the *pho* gene were of low quality and were all predicted proteins, therefore providing little information. However, using genomic homology comparisons from the UCSC Genome Browser (http://genome.ucsc.edu/) we were able to identify the syntenic region for four other fish species: *Takifugu rubripes* (fugu), *Tetraodon nigroviridis* (tetraodon), *Oryzias latipes* (medaka), and *Gasterosteus aculeatus* (stickleback). The genes flanking *phoenix* in zebrafish are *aspa* and *c11orf54* homologs. In all fish species examined, this synteny was maintained, but a synteny break occurred at this location in other vertebrate genomes. Located between *aspa* and *c11orf54* in all four fish species was a large predicted ORF. The corresponding predicted genes (GENSCAN00000016645 in fugu, chr14.906.1 in medaka, chrVII.1390.1 in stickleback and chr7.288.1 in tetraodon) had very weak but noticeable identity with the zebrafish *phoenix* gene. The overall identity of the fish homologues was still not significant (the best match was fugu to tetraodon at 35% identity in a limited region, with no other pair-wise comparison showing higher than 24% identity). The main feature of all proteins was a low complexity sequence with a large number of proline and lysine residues. Using ClustalW2 (http://www.ebi.ac.uk/Tools/clustalw2/index.html), we showed that the only significant identity in these predicted transcripts was in the amino-terminal end of the proteins (Fig .2D red box and 2C yellow rectangle). One small region was highly conserved in all transcripts (SDS-X(2-3)-SLF-[ILV]-TQ, red box in Figure 2D), which may represent a functional motif for this class of proteins. The high rate of divergence across the different fish species suggested that, the function of the protein can tolerate significant alterations in the primary sequence. Because these fish homologs were not identified through the usual BLAST comparisons, if homologs in other vertebrates exist, they will be difficult to find using traditional approaches. It is likely they will have to be identified through functional similarities instead of primary sequence homology.

To gather more information on the *phoenix* gene, we analyzed the putative phoenix product, looking for protein motifs and domains, using protein structure databases such as PFAM and CDD. We identified one putative functional domain using Motif Search (http://motif.genome.jp). It contained an abATPase signature, PSVHSPPSDS (P-[SAP]-[LIV]-[DNH]-X(3)-SXS), encoded in the first exon with the last serine overlapping the splicing site (Figure 2C). It is important to note, that evidence for the ATPase signature was not found in the other fish species, but those transcripts were computationally predicted and it is possible that there were missing exons or were incorrectly sequenced. In the largest *phoenix* splice form, there was a strongly predicted single membrane-spanning domain (Bioweb, Pasteur Paris, Figure 2C). Therefore, the majority of the protein appeared to be a very long, poorly structured, tail with a single potential anchorage point to a membrane. All the shorter gene products were predicted to be cytoplasmic. No secretion signal peptide was found (CBS Website, University of Denmark).

We performed *in situ* hybridization (ISH) on embryos from 8 hours post fertilization (hpf) through 5 dpf. The expression of the *phoenix* gene was first detected in the anterior and posterior lateral line system (Figure 2E) beginning at 2 dpf, and was uniformly expressed in all neuromasts and still maintained in this organ at 5dpf. *Phoenix* mRNA was found in a “ring-like” structure (Figure 2F, left panel), clearly staining the supporting cells, while it was absent from the center of the neuromasts, where hair cells were located (Figure 2F, right). Additionally, we found expression in discrete areas of the inner ear, which could correspond to the neuroepithelial patches (Figure S2B). The *pou4f3::GFP* line allowed us to observe in live larvae the hair cells in the inner ear which seemed unaffected by the mutation in wild-type (left) and mutant (right) cristae (Figure S2A)

Taken together, these observations clearly show that, we have identified a new gene with an upregulated expression in the supporting cells of the lateral line in zebrafish. Based on the various predictions, we speculate that Phoenix is most likely a structural protein, potentially carrying an enzymatic ATPase activity at its N-terminus.

### Impaired regeneration of hair cells in the lateral line of *phoenix* mutant larvae

**Figure 3:**
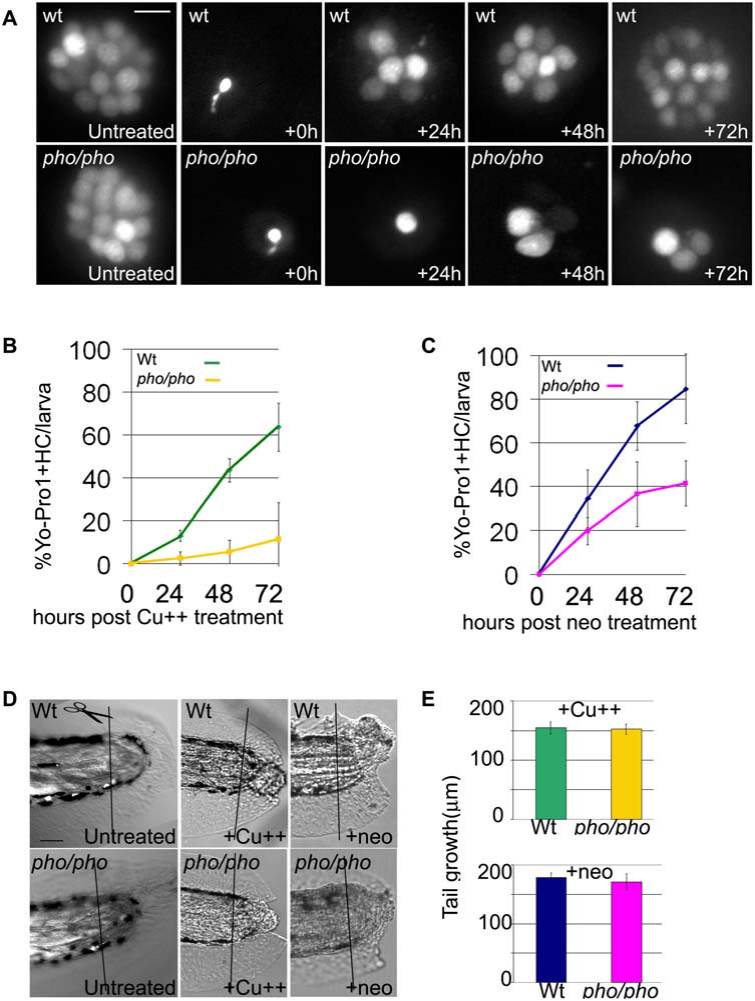
Lateral line hair cell regeneration is severely reduced in *phoenix* mutants but regeneration in other tissues is not affected. (A) Neuromasts of the lateral line are stained with YoPro 1, which exclusively stains hair cells. We treated wild-type (top panels) and *pho/pho* larvae (bottom panels) at 5dpf with copper (10mM). We monitored regeneration of hair cells over the following three days, at +24, +48, and +72h. As a reference, 5dpf wild-type and mutant neuromasts are shown just prior to treatment and just after the treatment (untreated and +0h, respectively, in the first and second columns from the left). (B) and (C) The number of hair cells was counted in untreated wild-type and mutant larvae at 5dpf (n = 39/53), 6dpf (n = 37/37), 7dpf (n = 23/22), and 8 dpf (n = 20/15). In each larva, we counted ten head neuromasts and all the tail neuromasts. We counted hair cells in healthy surviving larvae after 10 mM copper (B) or 200 mM neomycin (C) treatments. In (B), we counted wild-type (green line) and mutant (yellow line) larvae at +24h, (n = 40/36), +48h (n = 23/32), and +72h (n = 25/19), respectively, after copper treatment. In (C), we counted wild-type (blue line) and mutant (pink line) larvae at +24h, (n = 40/36), +48h (n = 23/32), and +72h (n = 25/19), respectively, after neomycin treatment. The numbers are presented as a percentage of regenerated hair cells in treated larvae, 100% being the respective number of hair cells at equivalent untreated wild-type and mutant stages. (D) We monitored the regeneration of tails in wild-type (top panels) and mutant (bottom panels) larvae. In untreated larvae (left panels), we amputated the tail at 3dpf and monitored the regeneration until 9dpf in wild-type (n = 8) and mutant (n = 20). We monitored the partial regeneration of tails amputated at 5dpf after copper (middle panels) and neomycin treatment (right panels). (E) Quantification of the tail regeneration after amputation at 5dpf after the copper treatment (upper graph), as measured in 8dpf wild-type (green, n = 11) and pho/pho (yellow, n = 10) larvae and after neomycin treatment (lower graph), as measured in 9dpf wild-type (dark blue, n = 6) and mutant (pink, n = 7) larvae. – 10 microns in (A) and 50 microns in (D).

Previous reports have demonstrated that copper [37] and neomycin, an antibiotic of the aminoglycoside family, effectively kill hair cells in the lateral line [38],[39],[41]. We treated 5dpf larvae with copper sulphate (10mM) for 2 hours or with neomycin (200mM) for 1 hour. After rinsing the larvae, we stained with Yo-Pro1, a vital dye that specifically accumulates in hair cells and binds irreversibly to DNA, allowing easy visualization of the hair cells in live larvae [38]. Untreated control wild-type (Figure 3A, left top panels) and *phoenix* mutant (Figure 3A, left lower panels) larvae at 5dpf showed a bright uniform staining of the hair cells, which adopt the shape of a rosette. Most hair cells were ablated immediately after the copper treatment (+0h) in all observed wild-type (top panel) and *phoenix* mutant (lower panel) larvae. At most, one remaining hair cell could be seen, but its irregular shape indicating that it was probably a dying cell (Figure 3A, second column). We concluded that we could efficiently destroy the lateral line hair cells with this treatment. Likewise, 4h after exposure to neomycin, we found that most hair cells were absent (data not shown). We next monitored, over the following three days, the reappearance of hair cells in wild-type and mutant larvae after both treatments (copper shown in Figure 3A, neomycin not shown). At one day post treatment (+24h, third column), we found an average of 6 stained hair cells in the wild-type (top panel), but at most one in the mutant (bottom panel) neuromasts. At two days post treatment (+48h, fourth column), we found on average 8 stained hair cells in the wild-type (top panel), but at most two in the mutant (bottom panel) neuromasts. Three days post treatment (+72h, fifth column), we found averages of 12 stained hair cells in the wild-type (top panel) vs. at most three in the mutant (bottom panel) neuromasts. Therefore we conclude that the regeneration process is severely impaired in *phoenix* mutant larvae.

We further quantified the treatments, counting Yo-Pro1 positive hair cells in 10 head and all trunk and tail neuromasts in wild-type and mutant larvae, after both copper and neomycin treatments and at all three time points during the recovery period. To factor in subtle developmental delays that we might have missed in the mutant, we counted untreated larvae at comparable stages. Numbers are presented as a percentage, 100% being the total number of hair cells present in untreated wild-type and mutant larvae at comparable stages. After copper treatment (Figure 3B) at all three time points, we found close to a five-fold difference in the number of Yo-Pro1 positive cells in wild-type when compared to *phoenix* neuromasts. After neomycin treatment (Figure 3C) we found nearly a two-fold difference at all stages. We conclude that in the absence of the *phoenix* product, neuromasts are not able to efficiently regenerate the destroyed hair cells.

### The regeneration defect in phoenix is specific to the lateral line

To determine if *phoenix*'s role in regeneration was specific to the lateral line, or represented a general inability to regenerate damaged tissues, we tested larval tail regeneration. We transected tails of 3dpf untreated wild-type (top panel) and mutant (lower panel) larvae and followed them to 9dpf (Figure 3D left panels). As described previously, the tails of wild-type larvae first closed the injured tips of the notochord and neural tube, followed by fin fold outgrowth [53]. Likewise, in mutant larvae, we observed a complete regeneration of the tail (Figure 3D, left lower panel). Furthermore, to exclude the fact that the treatments with the ototoxic agents could be interfering with the tail regeneration, we sectioned tails, immediately after copper (middle panels) or neomycin treatment at 5dpf (right panels). We monitored tail regeneration at 8dpf for copper and 9dpf for neomycin. While tail regrowth did not reach completion in wild-type or in mutant larvae, we saw no differences in the growth rate between wild-type and mutant larvae with either treatment. To further exclude more subtle differences, we quantified the tail growth after copper exposure (Figure 3E top graph) in 8dpf wild-type (green bar) and mutant (yellow bar) larvae and did not find a significant difference. Similarly, after neomycin treatment (Figure 3E, bottom panel), the growth of the tail in 9dpf wild-type (blue bar) and *phoenix* (pink bar) larvae was identical. Thus, we conclude that the *phoenix* mutation affects hair cell but not tail regeneration, indicating a defect specific to the lateral line.

Taken together, these observations strongly suggest that *phoenix* has an important role in the regeneration process of hair cells in the lateral line.

### The number of supporting cells is not affected by copper and neomycin treatments in mutant neuromasts

**Figure 4:**
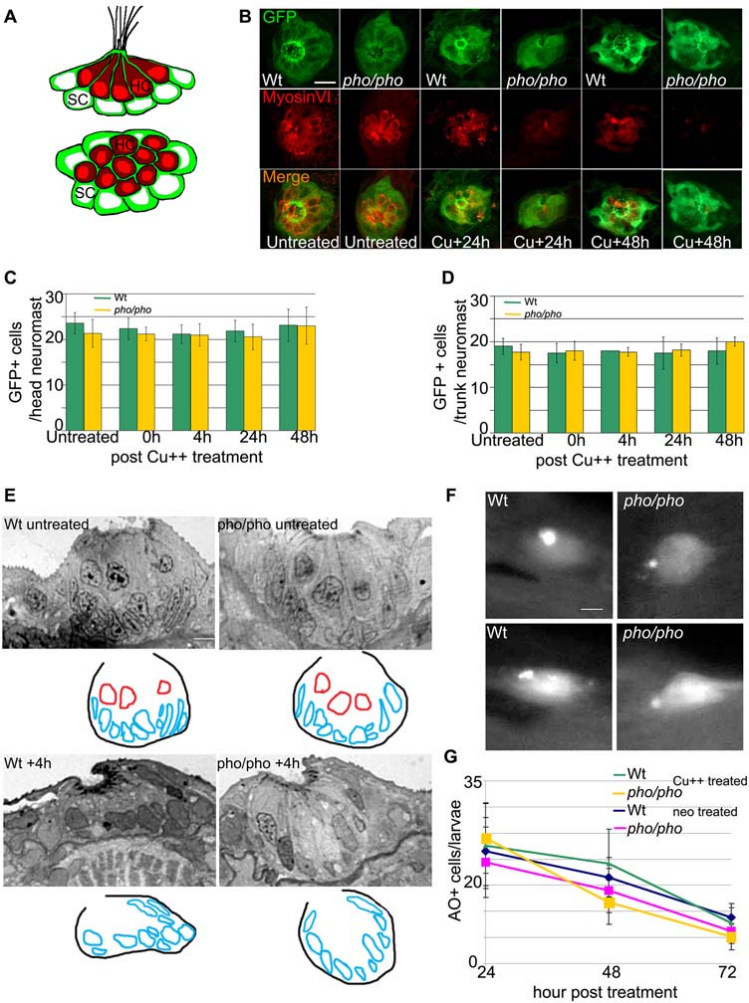
Supporting cells are present and do not die at a higher rate because of the treatments in the *phoenix* mutant neuromasts. (A) Schematic drawings of a side view (top panel) and a dorsal view (bottom panel) of a neuromast, illustrating the distribution of the two cell types. The hair cells (red) are centrally located and engulfed in a thin cytoplasmic furrow projected apically by the underlying supporting cells (green). (B) Immuno-labeled neuromasts with an antibody against GFP (top panels) staining the supporting cells and an antibody against Myosin VI (middle panels) staining the hair cells. Merged images are presented in the bottom panels. The SCM1 transgenic line, which we crossed into the *phoenix* mutant background, is expressing GFP exclusively in the supporting cells of the lateral line. We imaged transgenic wild-type (from the left, columns 1, 3, and 5) and transgenic *phoenix* mutant (columns 2, 4, and 6) larvae. Columns 1 and 2 are untreated 5dpf larvae. Columns 3 and 4 are larvae 24h post copper treatment. Columns 5 and 6 are larvae 48h post copper treatment. (C) Quantification of the number of GFP positive supporting cells respectively, in untreated (n = 18/21) and in +0h (n = 12/9), +4h (n = 15/17), +24h (n = 15/12), and +48h (n = 15/18) post copper treated transgenic wild-type (green bars) and transgenic *phoenix* mutant (yellow bars) head neuromasts. (D) Trunk neuromasts in wild-type and *phoenix* mutant transgenic larvae have been counted at the same stages, respectively untreated (n = 3/4), +0h (n = 4/3), +4h (n = 1/4), +24h (n = 2/5), and +48h (n = 2/3). (E) Semi-thin sections through wild-type (left panels) and mutant (right panels) neuromasts in untreated (top panels) and + 4h post neomycin treated (lower panel) 5dpf larvae. We used between 13 and 20 successive sections to reconstruct each wild-type and mutant neuromast (n = 1/2 untreated, n = 6/6 at +4h, respectively). We show one representative wild-type and mutant section at each stage. Under each section, a *camera lucida* sketch is showing the limits of the neuromast and the outlines of the nuclei (red for hair cells, blue for supporting cells). (F) Live images of two wild-type (left panels) and two mutant (right panels) neuromasts after AO stainings in larvae, 24h post copper treatment. (G) Scoring of the number of acridine orange (AO) positive cells in the lateral line/larvae, 24, 48, and 72h post copper (Cu++) or neomycin (neo) treatments. For the copper treatment, we monitored wild-type (green line) and mutant (yellow line, n = 22/13 at +24h, n = 14/12 at +48h, and n = 18/6 at +72h, respectively) larvae. For the neomycin treatment, we monitored wild-type (blue line) and mutant (pink line, n = 12/10 at +24h, n = 11/14 at +48h, and n = 10/11 at +72h, respectively) larvae. – 10 microns in (B) and (F) and 5 microns in (E).

The differences in regeneration capacity of hair cells in *phoenix* versus wild-type larvae could be due to a difference in susceptibility of the progenitor cells to the ototoxic agents used in our assays. There is substantial evidence pointing to supporting cells as the source of new hair cells in neuromast regeneration. The new hair cells are likely to arise from a population of dividing progenitor cells [42]–[44], however it is not clear if all or only a subset of the supporting cells represent cells capable of forming new hair cells [42]–[44]. In order to visualize all the supporting cells (including all the cells capable of initiating regeneration), we took advantage of a transgenic zebrafish line generated by an enhancer trap event, which expresses GFP in all supporting cells of the neuromast (MB/SB unpublished). We named this transgenic line SCM1 (Supporting Cell Marker 1). It was crossed into the *phoenix* mutant background in order to obtain mutant transgenic individuals. We evaluated the effect of either copper or neomycin treatment on the number of supporting cells in wild-type and mutant treated larvae after copper or neomycin exposure. We confirmed that there were no significant differences in the appearance of the supporting cells in the mutant transgenic larvae by performing the regeneration assay, followed by immuno-staining against GFP to detect supporting cells and anti-Myosin VI antibody to detect hair cells. In figure 4A, we present a schematic drawing, of the stainings in a transverse view (top) and a dorsal view (bottom) of a neuromast, as seen in both untreated wild-type and mutant neuromasts. We found centrally located hair cells (red), surrounded by the supporting cells (green) that form cytoplasmic furrows around hair cells, as described previously [54],[55]. In the control untreated wild-type (Figure 4B, fist column) and *phoenix* mutant (second column) neuromasts, hair cells were present and centrally located as visualized by anti-myosin VI antibody staining (red, middle and lower panels). The supporting cells, as expected, surrounded them as visualized with an anti-GFP antibody staining (green, top and lower panels). After copper (shown in Figure 4B) and neomycin (not shown) treatments, we could see a clear difference between wild-type and mutant neuromasts in the number of hair cells (red, middle and lower panels) at all stages of recovery, +24h (columns 3 and 4), +48h (columns 5 and 6) and +72h (not shown). Importantly, we did not see an obvious difference at any of the observed stages post-treatment, in the number or appearance of supporting cells (green, top and lower panels) in *phoenix* mutants compared to wild-type larvae. This finding suggests that, while hair cell regeneration is greatly decreased in *phoenix* mutant neuromasts, the number of supporting cells appear unaffected.

To further support this finding, we used confocal imaging to count supporting cells in the immuno-stained larvae (Figure 4C and 4D) in wild-type (green bars) and *phoenix* mutants (yellow bars). We counted the supporting cells in 4 different neuromasts in the head (Figure 4C) and in two different trunk neuromasts (Figure 4D) in each larva. Cell numbers were determined for untreated or copper treated larvae at +0h, +4h, +24h and +48h after the treatment. We did not find significant differences in any of the observed stages between wild-type and mutant larvae.

Another possible explanation for the observed phenotype is that mutant progenitor cells are dividing but that the newly formed precursor cells do not survive because they are hyper-sensitive or unstable after the chemical exposure. To detect dying cells in the supporting cell layer in the mutant neuromasts, we analyzed semi-thin sections (n = 2/2 wild-type and mutant larvae, respectively in Figure 4E). We reconstructed each neuromast using between 12 and 20 successive sections. We show representative examples of a wild-type (left panels) and a mutant (right panels) neuromast, untreated (Figure 4E, top panels) and 4 hours after neomycin treatment (lower panels). Before and after treatment, wild-type and mutant neuromasts were indistinguishable. As outlined in the *camera lucida* drawings under each section, the wild-type neuromast had three hair cells (nuclei outlined in red) and ten supporting cells visible (nuclei outlined in blue). In the mutant neuromast, three hair cells and ten supporting cells were visible. Four hours after neomycin treatment hair cells were completely destroyed in the wild-type and in the mutant neuromast (Figure 4E, lower panels). The supporting cells appeared unaffected in both wild-type and *phoenix* mutant neuromasts. Ten nuclei in the wild-type and ten in the mutant neuromast were visible (as outlined in the *camera lucida* in blue). Thus, at all observed stages, we did not notice a significantly higher cell death rate among the supporting cell population in *phoenix* mutant larvae.

Next, we examined cell death in live larvae using acridine orange [37]. We stained larvae at +24h, +48h and +72h post treatment. Dying cells displayed strong staining as shown in two wild-type (Figure 4G left panels) and two mutant (Figure 4G right panels) neuromasts at +24h post copper treatment. We counted the acridine orange positive cells in ten head and all trunk and tail neuromasts (Figure 4F) after copper treatment in the wild-type (green line) and mutant (yellow line), or after neomycin treatment (wild-type: blue, mutant: pink). At all stages observed we did not find a significant difference. Additionally, we looked at cell death in the neuromasts, using TUNEL stainings and did not find any obvious difference in the rate of dying cells at all stages observed (data not shown).

We therefore conclude that the copper and neomycin treatments do not significantly affect the number of supporting cells in either wild-type or mutant larvae. Moreover, we rule out the possibility that progenitor cells may be dying in *phoenix* neuromasts as we did not observe an increase in cell death rates in mutant larvae compared to wild types. Taken together these results indicate that the regenerative capacity rather than the survival of supporting cells is affected by the *phoenix* mutation.

### Supporting cell proliferation is impaired during regeneration in *phoenix* mutant neuromasts

**Figure 5:**
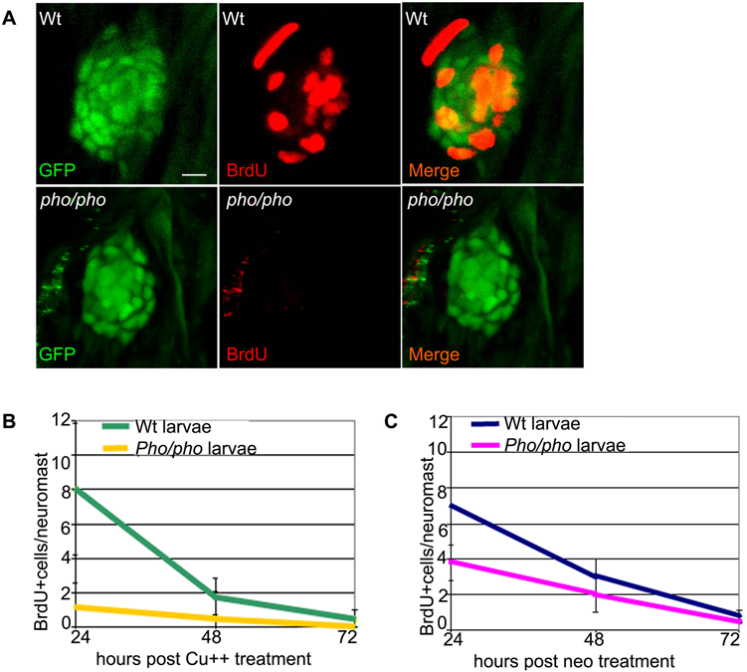
Supporting cells do not divide as efficiently after hair cell damage in the *phoenix* mutant neuromast. (A) BrdU staining (red, in the middle and right panels) in the *SCM1::GFP* transgenic wild-type (top) and the *SCM1::GFP* transgenic *phoenix* mutant neuromast (bottom) of recovering larvae (n = 20/20), 24h after copper treatment. The supporting cells are counterstained with GFP antibodies (green in left and right panels). (B) Quantification of the BrdU positive cells/neuromast in wild-type (green line) and mutant (yellow line) recovering neuromasts, at +24h (n = 11/10), +48h (n = 10/6), and +72h (n = 7/6) after copper treatment. Larvae are exposed to BrdU for 6h prior to being sacrificed. (C) Quantification of the BrdU positive cells/neuromast in wild-type (blue line) and mutant (pink line) recovering larvae, at +24h (n = 22/40), +48h (n = 20/33), and +72h (n = 13/32) after neomycin exposure. – 10 microns in (A).

Since the number of supporting cells in mutant neuromasts is not unlike that of wild type neuromasts, we turned our attention to differences in cell division to explain the regeneration phenotype observed in *phoenix* larvae. To test the hypothesis that the *phoenix* mutation is affecting proliferation of the progenitor cell population, we exposed SCM1 transgenic wild-type and mutant larvae, treated with copper or neomycin, to BrdU for 6h prior to fixing them, at +24h, +48h and +72h after chemical treatment. We subsequently double-stained the larvae with an anti-BrdU antibody (red in Figure 5A, middle and right panels) and with an anti-GFP antibody (green in left and right panels). At +24h after copper (Figure 5A) and neomycin (not shown) treatments, the BrdU-labeled transgenic wild-type (Figure 5A, top panels) and the transgenic *phoenix* mutant (lower panels) larvae showed a striking difference in the number of BrdU positive cells. On average, we found eight BrdU positive cells in the transgenic wild-type neuromasts, in mutant neuromasts we typically found no positive cells while at most we found two. Thus the number of BrdU positive cells in the progenitor cells population in the mutant neuromasts is drastically reduced, most likely explaining the decrease in the number of newly regenerated hair cells in *phoenix* mutant embryos.

To obtain quantitative data to support the above conclusion and to follow the dynamics of BrdU incorporation over time, we counted BrdU positive cells after copper treatment (Figure 5B) in at least ten wild-type (green line) and ten *phoenix* mutant (yellow line) neuromasts per larva. BrdU was added to the medium 6h before fixation allowing us to determine rates of DNA synthesis in equivalent time-windows during the 3 days regeneration period. Since recovery to a normal number of hair cells is reached at around 72 hours after treatment, this time point represents the rate of BrdU incorporation in undamaged neuromasts, or the BrdU baseline. At +24h of recovery, we had an eight-fold difference in the number of BrdU positive cells between wild-type and mutant neuromasts. This difference was still two-fold at +48h of recovery and both returned to the base line frequency at +72h. We performed similar tests after neomycin treatment (Figure 5C, wild-type: blue line and mutant: pink line). At +24h, we found close to a two-fold difference of BrdU labeled cells, and at +48h a 33% decrease between wild-type and mutant neuromasts.

Taken together, these results show that the lack of the Phoenix protein has a significant negative impact on proliferation in the regenerating neuromast, which is not due to a reduction in the number of supporting cells. Thus, *phoenix* has an important role in ensuring proper proliferation of the supporting cells during hair cell regeneration in neuromasts.

## Discussion

We have isolated a mutation in a previously uncharacterized gene in zebrafish that we have named *phoenix* because of an observed deficiency in the regeneration of the hair cells of the lateral line. Identical phenotypes were observed in two different alleles, caused by independent retroviral integrations in the *phoenix* gene in different genetic backgrounds. This unambiguously links the phenotype to this particular gene, which encodes a rapidly evolving protein with no obvious homologs in the current genomic databases. The predicted Phoenix protein is a long peptide of low complexity with a predicted ATPase domain reminiscent of many cytoskeletal proteins. This would argue in favor of a structural protein, possibly interacting with the cytoskeleton and subcellular membranes. How it is linked to the ability of the supporting cells to regenerate will require further investigation. It also remains to be discovered whether mammalian cells have a similar molecule; we were unable to detect a related sequence in the mammalian genomes. Interestingly, inner ear hair cells in mammals are unable to regenerate from supporting cells postnatally and, therefore, exploring the molecular differences between supporting cells between mammals and other vertebrates becomes of interest when trying to understand differences in regenerative capacity.

We show that the expression of the *phoenix* gene is strongly upregulated in the supporting cells of the lateral line and that the lateral line morphology is essentially normal in the *phoenix* mutant larvae at all stages observed, from 2dpf to 12dpf. However it is important to note that, because we did not assess a possible maternal contribution for this particular gene, we cannot definitively exclude a potential role for *phoenix* during early development in general or of the lateral line in particular.

Nevertheless, our regeneration assay performed in larvae between 6 and 8dpf, clearly shows that the number of regenerated hair cells is reduced by five-fold after copper treatment and by two-fold after neomycin treatment in *phoenix* mutant neuromasts. Therefore, the zygotic expression of the *phoenix* gene is clearly crucial for proper regeneration of the hair cells in the lateral line. Observations at later stages never showed full recovery to a wild-type number of hair cells in the mutant neuromasts, demonstrating that the deficit in the regenerative process is irreversible. We have also found expression of *phoenix* in the neuroepithelia of the inner ear, but unfortunately regeneration of the hair cells in the ear cannot be assessed with our assay, as water-borne ototoxic compounds do not diffuse into the otic capsule.

We further show that the regeneration deficiency is specific to the lateral line, as we did not see a similar effect during tail regeneration. In combination with the restricted expression of the gene in supporting cells of the lateral line, this strongly suggests that, zygotic *phoenix* is involved specifically in the regeneration of hair cells in the lateral line, independent of the ototoxic treatment (copper or neomycin) used.

It is intriguing that while the two treatments trigger similar responses, they are significantly different in the amplitude of the elicited effect. The reason there is a nearly 2.5 times greater inability of the supporting cells to respond properly after copper compared to neomycin treatments, is not clear. However, it is to be noted that the wild-type regeneration is also significantly slower (about two times) after the copper treatments when compared to neomycin treatments, as we found and as it has been described previously [37],[43]. One possibility, as has been suggested previously [44], is that there is more than one mechanism of regeneration occurring in the supporting cells. The supporting cells probably do not represent a uniform population. For instance, one type of supporting cells, the “early responders” may be unaffected in the case of hair cells killed with neomycin, but might be more severely affected by the copper treatment leaving them unable, either temporarily or permanently, to respond and enter division. In less mature embryos (3dpf), the concentration of copper used (10 µM) has been shown to eliminate post-mitotic precursors, thus requiring cell division and longer times for regeneration when compared to lower doses of the metal [37].

Additionally, it was recently shown in a screen to identify genes that modify hair cell resistance in the lateral line, that different ototoxic agents can elicit different pathways for protection and survival of hair cells in treated neuromasts [56]. It is therefore also possible that the regenerative response in supporting cells depends partially on the type of ototoxic agent applied. Namely, the trigger or the response to the trigger of regeneration in supporting cells could rely on different pathways, partially overlapping or complementing each other depending on the ototoxic chemical. Further investigation will allow us to distinguish between these possibilities. It appears that *phoenix* is upstream of the differential response between neomycin and copper, as the differences in the rates of response in mutant larvae remain similar to the differences seen in wild-type larvae.

We showed that the death rate of supporting cells was unaltered in mutant neuromasts as observed by three different approaches (histology, acridine orange and TUNEL staining). Furthermore, making use of the MSC1 transgenic line that expresses GFP exclusively in the supporting cells, we demonstrated no loss in the number of those cells in mutant neuromasts. It is to be noted that the counting of cells in the neuromast is technically challenging. Although we did not see obvious differences, we cannot exclude a possible subtle reduction in the number of supporting cells. If a key subset of cells critical to the initiation of regeneration is missing, even a subtle difference in number might have a drastic effect on regeneration, which we cannot exclude at our level of analysis. However, it was previously shown that the regeneration process is happening in many supporting cells simultaneously, encompassing most of the neuromast [44] making this possibility unlikely. We clearly show that a significant number of supporting cells are present and that the *phoenix* mRNA is expressed in all supporting cells, therefore the most likely explanation is that the regenerative potential of all supporting cells is severely impaired.

To assess proliferation of the progenitor cells, we follow the incorporation of BrdU during the S-phase of the cell cycle in neuromasts. The incorporation of BrdU is detected in cells that have exited the cell cycle and have differentiated. Therefore, it provides no information on the post-mitotic state of the BrdU positive cells. However, as the number of regenerated hair cells correlates closely to the number of BrdU labeled cells and as we do not see additional cell death in either wild-type or mutant neuromasts, this would suggest that the progenitor cells that actually enter S-phase proceed through the entire cell cycle and into differentiation. In the *phoenix* mutant larvae, this regenerative process is happening in a significantly reduced number of supporting cells. Further work is required to address the timing of the blockage occurring in the supporting cells in mutant neuromasts, but it is clearly occurring before DNA synthesis initiates. This invaluable information will help to elucidate the mechanism of action of the novel *phoenix* gene in the regeneration process of hair cells in the lateral line.

## Materials and Methods

All animals were handled in strict accordance with good animal practice as defined by the relevant national and/or local animal welfare bodies, and all animal work was approved by the appropriate committee (NHGRI protocol # G-01-3).

### Fish care and husbandry

Fish care and husbandry were performed according to [57] in compliance with NIH guideline for animal care. The *hi43* allele of *phoenix* was recovered in a retroviral screen performed at MIT [58]. The genomic locus of the retroviral integration and the putative cDNA were determined as described in [45]. Genotyping was done with primers GGAGATCGACAGCGCCCTGAAG and AAACTGCTGAGGGCTGCTGGACCGCATC. The *zm* allele (ZM_00003486) was purchased (Znomics, Inc) and carriers were genotyped using the primers CGAGACCCCGCCGCCTGATGTT and GACGCAGGCGCATAAAATCAGTC. The MSC1 transgenic line was a gift from B. Weinstein and carriers were identified by detecting specific expression of GFP in the lateral line. The *cldnB::GFP* line [46] was obtained from M. Allende, the *pou4f3::GFP* line [47] from H. Baier and the *ET20::GFP* line [48] from Vladimir Korzh.

### Staining with vital dyes (YoPro-1, Acridine Orange, and FM1-43) and treatment with copper or neomycin of live larvae

All active agents were added to system water at 28°C. During the staining/treatment, larvae were kept in cell strainers (BD Falcon) in 6 well plates (Costar, Corning, Inc)) at a maximum of 35 larvae/well. This allowed rapid transfer of the larvae during the post-treatment rinsing. All larvae were rinsed 6×3 minutes with system water and then transferred into large Petri dishes with abundant fresh system water. After the various treatments, only healthy looking larvae displaying no additional morphological or behavioral defects were kept for subsequent analysis over the following 72 hours. The number of hair cells and the AO positive cells were counted in 10 head and all the trunk and tail neuromasts, in each larva.

We added **YoPro-1** (Molecular probes) for 15 minutes at 2mM, as described in [38], **FM1-43** (Molecular probes) for 1minute at 2mM, as described [49] and **Acridine orange** (AO) hemi (zinc chloride, Sigma) for 5 minutes at 2mg/ml, as described [37]. **Copper** (Copper(II) sulfate, Sigma) was added for 2h at 10mM as described in [37] and **Neomycin** (sulfate, Calbiochem) for 1h at 200mM as described in [38],[39]. Monitoring, counting and imaging of the lateral line in live larvae, after using the different life dyes, was done on an inverted Zeiss AXIOVERT200M equipped with an Apotome Grid Confocal. Larvae were anesthetized with MS222 (0.005%) and mounted on a cover slip in 2% Methylcellulose (Sigma).

### Tail transections

Five day old larvae were allowed to recover for an hour after copper or neomycin treatment. Subsequently, larvae were anesthetized with 0.005% MS-222 (Sigma) and placed on a clean glass slide and a portion of their tail was amputated with a scalpel. We cut not just the fin, but also a portion of the actual tail including the neural tube, the notochord, and the somites, to evaluate the regeneration of other tissues in addition to the fin itself. Three day old untreated larvae were similarly processed. To follow and image the tail growth over 3 to 6 days post amputation, we mounted anesthetized larvae on cover slips in 2% methylcellulose. Measurements were made on pictures taken from live larvae on the inverted Zeiss AXIOVERT200M, using the Axiovision software from Zeiss. The region of amputation was usually easily identifiable, as it presented healing scars, allowing a reasonably accurate measurement of the newly grown tail.

### BrdU treatment

BrdU (Sigma) was diluted to 10mM in system water and larvae were exposed for 6h before fixation at the chosen time points (24, 48 and 72 hours post treatment) o/n with 4% formaldehyde (Electron Microscopy Sciences) in 1× PBS (Quality Biological, Inc.). Double staining was done as described [40],[44], with a fluorochrome labeled mouse monoclonal antibody against BrdU (Molecular probes) and a fluorochrome labeled rabbit polyclonal antibody against GFP (Abcam). Larvae were mounted on slides in Aquapolymount (Polyscience, Inc) and at least 10 neuromasts/larva were counted. The imaging was done on an upright confocal microscope (Zeiss AXIOVERT)

### Immunofluorescence on larvae

Untreated and copper treated larvae were fixed o/n with 4% formaldehyde (Electron Microscopy Sciences) in 1× PBS (Quality Biological, Inc.), at various stages of interest and subsequently stored in 100% methanol. After progressive rehydration (25%, 50% 75% and 100% PTW (PBS1×, 0.001% Tween and 0.001% DMSO)), larvae were treated with acetone for 7mn at −20°C. Subsequently, we rehydrated and rinsed them 3×5mn in PTW. Next we digested them with 1mg/ml collagenase (Sigma) in PTB (PTW + 10% goat serum +10% BSA) for 35mn. After 5×5mn rinses with PBT, we pre-incubated the larvae 4 hours in PBT. Larvae were incubated o/n with the polyclonal rabbit primary antibody (1/200) against Myosin VI (Proteus Biosciences, Inc) and a fluorescently labeled monoclonal mouse primary antibody (1/200) against GFP (Abcam). The next day we rinsed the larvae 6×10 mn in PTW and preincubated them again for 4 hours in PBT. The fluorescently labeled secondary anti-rabbit antibody (1/500) was added o/n. The next day 6×10 mn rinses were performed. Larvae were mounted on slides in Aquapolymount (Polyscience, Inc) for imaging on an upright confocal microscope (Zeiss AXIOVERT).

### Semi-thin and ultra thin sections for TEM on zebrafish larvae

Larvae were fixed overnight at in 2.5% glutaraldehyde (Sigma) and 4% paraformaldehyde prepared from paraformaldehyde (Sigma) in 0.1M sodium cacodylate buffer (Sigma). Larvae were then rinsed and post-fixed 1h at room temperature in reduced osmium (1∶1 mixture of 2% aqueous potassium ferrocyanide) as described previously [59]. After post-fixation the cells were dehydrated in ethanol and processed for Epon (Sigma) embedding. Semi-thin sections (300 nm) were cut and collected on a glass slide, and subsequently stained using toluidine blue (Sigma). The analysis and imaging were done on an inverted Zeiss Axiovert200M. Ultra thin sections (80 nm) were cut on a Reichter-E ultramicrotome, collected on copper grids and stained with lead citrate (Sigma) for 2 min. Sections were then examined with a CM 10 Philips electron microscope at 80kV.

### Whole mount *in situ* hybridization

Performed as described previously [60]. We designed an antisense probe, using the following primers TCAACTGATGTATTTCCTGGGC and GTTTTGCTCCACTATCTGACCTTT. The probe was hybridized at 62°C. Larvae were mounted on slides in Aquapolymount (Polyscience, Inc) for imaging on an upright confocal microscope (Zeiss AXIOVERT).

### Isolation of the BAC containing the *phoenix* gene and bioinformatic analysis

We screened the zebrafish BAC library (Chori 211, BacPac consortium) with a probe generated from total RNA (5 dpf larva), with the primers AGATCTTGAGATTGCCGAATGT and CATCTCTCTCACCTTCTTCAGTGAC. The BAC was sequenced, by shotgun assembly and brought to finished quality (≤1 error per 50kb) at the National Intramural Sequencing Center (NISC). Sequences were aligned to the zebrafish genome (UCSC Genome Browser http://genome.ucsc.edu/) and syntenic regions identified using genomic chain comparisons in *Takifugu rubripes* (fugu), *Tetraodon nigroviridis* (tetraodon), *Oryzias latipes* (medaka), and *Gasterosteus aculeatus* (stickleback). Genescan or N-SCAN [50] predicted transcripts between the two flanking genes were identified and compared using ClustalW [61] (http://www.ebi.ac.uk/Tools/clustalw2/index.html). The BLOSUM protein matrix [62] was used with a window of 7 and gap penalty of 2.

### RT-PCR, sequencing, and cDNA identification

We prepared total RNA extracts from embryos and Pac2 cells (which is an embryonic zebrafish cell line described in [51] in Trizol (Invitrogen). For the RT-PCR, we retro-transcribed specific cDNA using superscript II Reverse transcriptase (Invitrogen), following the manufacturer's protocol. For the cloning of all transcripts shown in Figure 2B and 2C, we used the same set of 2 nested forward primers CCATATCAAAACAGAGCTGTGCTAC and GTCAAGGCAGAGTAAGCAAGTGACACTG, both located in the 5′UTR, respectively starting 142bp and 121bp upstream of the start codon. For the cloning of transcripts 2, 3 and 6, they were respectively coupled with the reverse primers GTTCTGTTTTCTTTTCCTTGTCAACGCC and CCTTCTCTCCCTGATAAAGTCTGGCAACC both found in the 5′end of exon 7. For the cloning of the transcript 1, we coupled them respectively with the reverse primers CATTCATTCATTCATTCATTTTCCT and TTTCCTTTTGCTTAGTCCCTTATTT both found in the 3′end of exon 2. For the cloning of the transcript 4 we coupled them respectively with the reverse primers TTTTACATCTACAGAATCGTGAAAAA and AATTGTGATTCTCATTTTAGCCAGA, both found in the 3′ end of exon 6. For the cloning of the transcript 5 we coupled them respectively with the reverse primers TTTTTCTTTCATAGCGAACACAAAG and TTTAAACTGTCAGCTCTTGATGC, both found in 3′end of exon 5. To verify the quality of the Total RNA from different stages, which we used for the RT-PCR, we amplified a 159bp fragment of b-actin using the following primers, GACCCAGACATCAGGGAGTGATGG and AGGTGTGATGCCAGATCTTCTCCAGT. All sub-cloning for subsequent sequencing were done using the TOPO TA cloning Kit (Invitrogen), following the manufacturer protocol. Sequencing was performed by ACGT, Inc. The analysis of the sequences was done using the Sequencher (Gene Codes Corp.) program, with the genomic sequence of the *phoenix* gene (as previously isolated in the BAC) as a reference.

### Statistical analysis

At all time points and in all graphs, the p values for wild-type versus mutant larvae were calculated using a Student's T test (two tailed) with two samples of equal variance and considered significant when p was <0.05. Error bars in the different graphs represent the standard deviation, or standard error, depending on the number of samples (n) analyzed.

### Image reconstruction

Stacks of images collected on the inverted microscope were treated with the Axiovision software from Zeiss. Stacks of images collected on the confocal microscope were treated with the LSM software from Zeiss. All images or stack of images were exported as tiff files, which were subsequently processed with Photoshop.

## Supporting Information

Figure S1 Live imaging of transgenic lines crossed into the *phoenix* background show no differences from wild-type larvae. A. We crossed the *phoenix* mutant line into the *cldnB::GFP* transgenic background, which expressed GFP (green in first and third panels) in all cells of the neuromast. We stained untreated 5 dpf larvae with FM1-43 (red in second and third a panel). Shown are images of live wild-type (top panels) and *phoenix* (bottom panels) neuromasts. A *camera lucida* drawing of each merged picture is highlighting the GFP positive cells contours (green) and the FM1-43 positive cells (red) in the fourth panel. B. We crossed the *phoenix* mutant line into the *pou4f3::GFP* transgenic background, which expressed GFP in all of the neuromast's hair cells. Dorsal views (left panels) and lateral views (right panels) of wild-type (top panels) and *pho/pho* (bottom panels) are shown. A *camera lucida* drawing of each panel is outlining the GFP positive hair cells of each respective 41 panel. C. We crossed the *phoenix* mutant into the *ET20::GFP* transgenic background, which expressed GFP in a subset of supporting cells known as the mantle cells. Two representative examples of wild-type (top panels) and of *phoenix* mutant (bottom panels) neuromasts are shown. A *camera lucida* drawing of each panel is outlining the GFP + cells of each respective panel. - 10 microns in all panels.
(6.66 MB TIF)

Figure S2 Hair cells seem unaffected in the *phoenix* mutant ear. Wild-type expression of the *phoenix* mRNA and protein. A. Live images in *pou4f3;;GFP* transgenic animals of hair cells in a crista of a wild-type (left panel) and a phoenix mutant (right panel) ear. B. *In situ* hybridization with an antisense probe against *phoenix* in the inner ear of a 4dpf old embryo (top panel). A *camera lucida* drawing (lower panel) shows the positions of the two otoliths (red), of the cristae's (light green) and the maculae's (dark green) hair cells. The AP/DV ventral orientation is indicated. C. Immunofluorescence on cultured zebrafish cells (Pac2) using EX1 (green in the top and bottom left panels) or EX7 (green in the top and bottom right panels) antibodies. To highlight the ER, we co-stained with an antibody against PDI (ER-associated protein disulfide isomerase, red in middle and bottom left and right panels). DAPI was used to counterstain nuclei (blue in the two bottom left and right panels). Merged images (bottom, left and right panels) showed a cytoplasmic staining of phoenix, which was more concentrated in the perinuclear region. It appeared excluded from the ER, as there was no co-localization with DPI. Note how phoenix protein was upregulated in cells preparing to enter division (white arrow in the top right panel). D. Western blot on PAC2 cell extracts with two rabbit polyclonal antibodies, raised against an epitope encoded by exon 1 (EX1) or by exon 7 (EX7). A common band in the range of 95KDa was found with both antibodies (black arrow). - 10 microns in A, 50 microns in B and 3 microns in C.
(3.51 MB TIF)
