# Supplementary figures and images for: Phoenix Is Required for Mechanosensory Hair Cell Regeneration in the Zebrafish Lateral Line
Source: PLoS Genet. 2009 Apr 17;5(4):e1000455. doi: 10.1371/journal.pgen.1000455 (PMC2662414; doi:10.1371/journal.pgen.1000455)

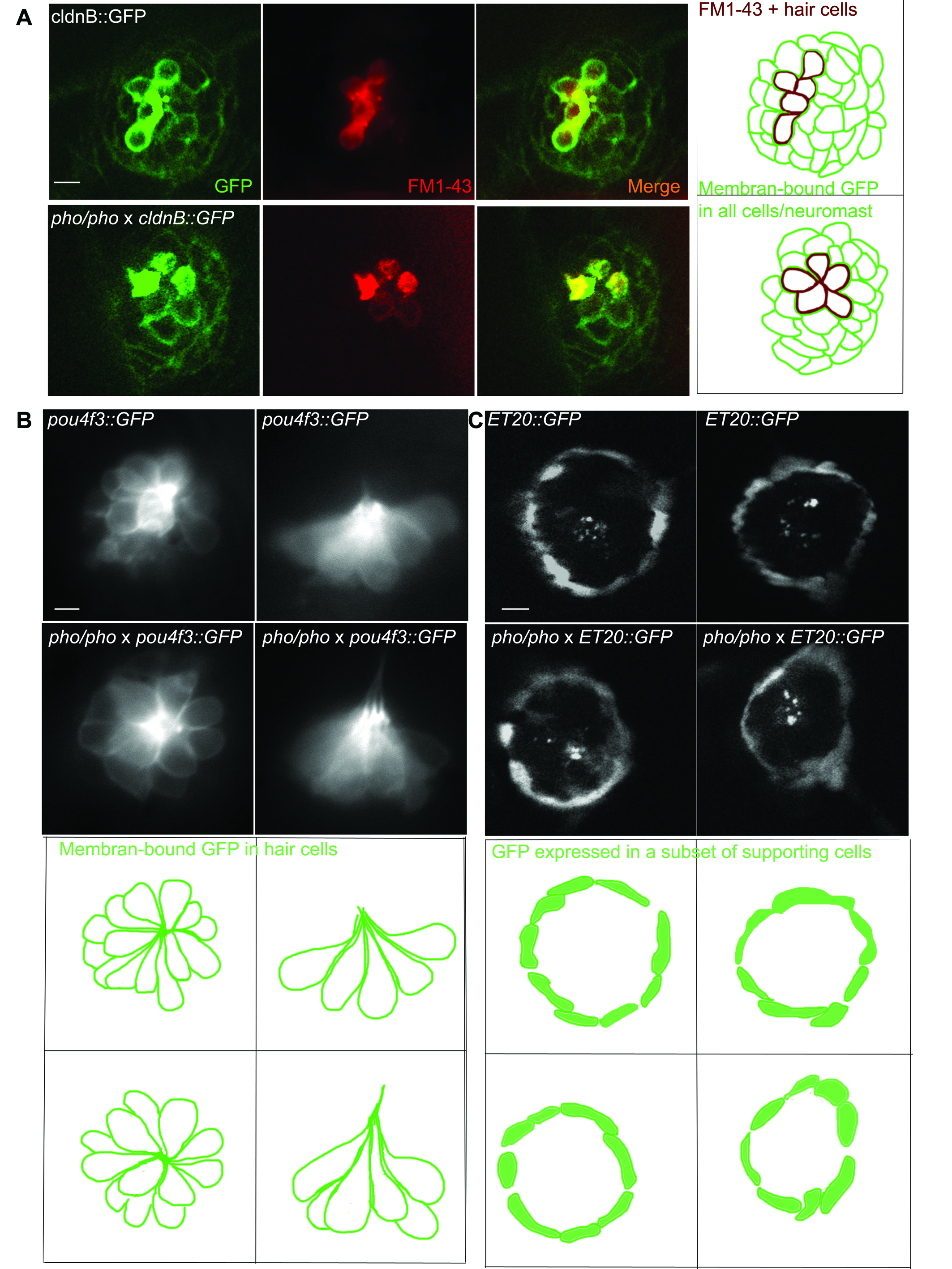

Supplement: Figure S1 — Live imaging of transgenic lines crossed into the phoenix background show no differences from wild-type larvae. A. We crossed the phoenix mutant line into the cldnB::GFP transgenic background, which expressed GFP (green in first and third panels) in all cells of the neuromast. We stained untreated 5 dpf larvae with FM1-43 (red in second and third a panel). Shown are images of live wild-type (top panels) and phoenix (bottom panels) neuromasts. A camera lucida drawing of each merged picture is highlighting the GFP positive cells contours (green) and the FM1-43 positive cells (red) in the fourth panel. B. We crossed the phoenix mutant line into the pou4f3::GFP transgenic background, which expressed GFP in all of the neuromast's hair cells. Dorsal views (left panels) and lateral views (right panels) of wild-type (top panels) and pho/pho (bottom panels) are shown. A camera lucida drawing of each panel is outlining the GFP positive hair cells of each respective 41 panel. C. We crossed the phoenix mutant into the ET20::GFP transgenic background, which expressed GFP in a subset of supporting cells known as the mantle cells. Two representative examples of wild-type (top panels) and of phoenix mutant (bottom panels) neuromasts are shown. A camera lucida drawing of each panel is outlining the GFP + cells of each respective panel. - 10 microns in all panels. (6.66 MB TIF) [file pgen.1000455.s001.tif]

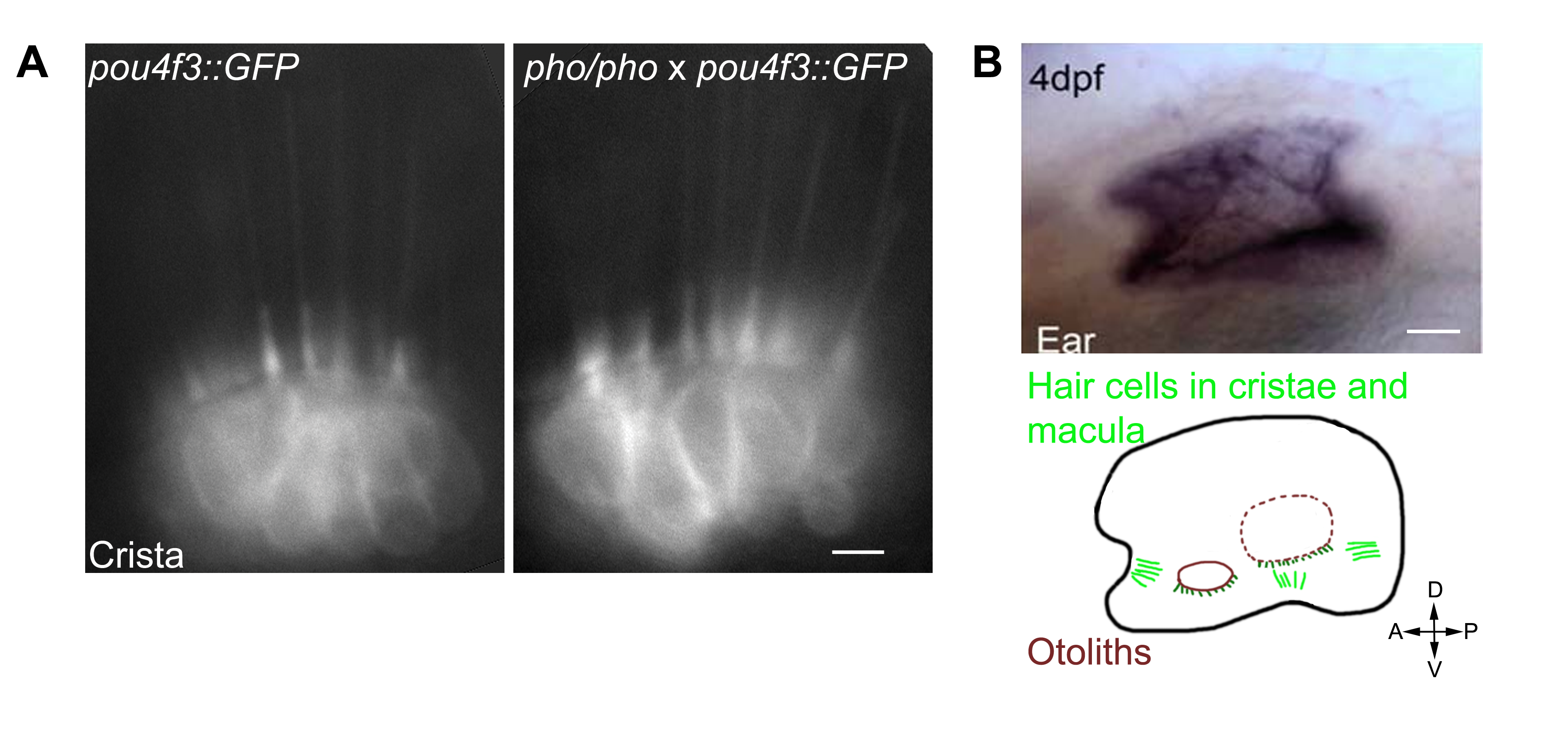

Supplement: Figure S2 — Hair cells seem unaffected in the phoenix mutant ear. Wild-type expression of the phoenix mRNA and protein. A. Live images in pou4f3;;GFP transgenic animals of hair cells in a crista of a wild-type (left panel) and a phoenix mutant (right panel) ear. B. In situ hybridization with an antisense probe against phoenix in the inner ear of a 4dpf old embryo (top panel). A camera lucida drawing (lower panel) shows the positions of the two otoliths (red), of the cristae's (light green) and the maculae's (dark green) hair cells. The AP/DV ventral orientation is indicated. C. Immunofluorescence on cultured zebrafish cells (Pac2) using EX1 (green in the top and bottom left panels) or EX7 (green in the top and bottom right panels) antibodies. To highlight the ER, we co-stained with an antibody against PDI (ER-associated protein disulfide isomerase, red in middle and bottom left and right panels). DAPI was used to counterstain nuclei (blue in the two bottom left and right panels). Merged images (bottom, left and right panels) showed a cytoplasmic staining of phoenix, which was more concentrated in the perinuclear region. It appeared excluded from the ER, as there was no co-localization with DPI. Note how phoenix protein was upregulated in cells preparing to enter division (white arrow in the top right panel). D. Western blot on PAC2 cell extracts with two rabbit polyclonal antibodies, raised against an epitope encoded by exon 1 (EX1) or by exon 7 (EX7). A common band in the range of 95KDa was found with both antibodies (black arrow). - 10 microns in A, 50 microns in B and 3 microns in C. (3.51 MB TIF) [file pgen.1000455.s002.tif]
